# Study on the Durability of Graphene Oxide–Nanosilica Hybrid-Modified Sticky Rice–Lime Paste

**DOI:** 10.3390/nano15151194

**Published:** 2025-08-05

**Authors:** Ke Li, Donghui Cheng, Yingqi Fu, Xuwen Yan, Li Wang, Haisheng Ren

**Affiliations:** 1 School of Civil Engineering and Transportation, Northeast Forestry University, Harbin 150040, China; likelike@nefu.edu.cn (K.L.); nefuff@nefu.edu.cn (Y.F.); nefuyxw@nefu.edu.cn (X.Y.); wldxy@nefu.edu.cn (L.W.); 2 School of Architecture and Civil Engineering, Qiqihar University, Qiqihar 161006, China; 3 Intelligent Transportation System Research Center, Southeast University, Nanjing 211189, China; ren_hs510@seu.edu.cn

**Keywords:** sticky rice–lime paste, graphene oxide, nano-silicon dioxide, durability, restoration of ancient buildings

## Abstract

In order to improve the durability performance of sticky rice–lime paste in ancient masonry restoration materials, the effect of graphene oxide–nanosilica hybrids (GO–NS) on its basic physical properties and durability performance was investigated. The surface morphology, physical phase characteristics and infrared spectra of GO–NS and its sticky rice–lime paste were analysed by SEM, FE-TEM, XRD and FTIR. It was shown that NS successfully attached to the GO surface and improved the interlayer structure of GO. GO–NS reduces the fluidity and shrinkage of sticky rice–lime paste, prolongs the initial setting, shortens the final setting and significantly improves the compressive strength, water resistance and freeze resistance. As NS improves the interlayer structure of GO, it provides nucleation sites for the hardening of the sticky rice–lime paste, improves the quantity and structural distribution of the hardening products and reduces the pores. The NS undergoes a hydration reaction with Ca(OH)_2_ in the lime to produce calcium silicate hydrate (C–S–H), which further refines the internal pore structure of the sticky rice–lime paste. As a result, the GO–NS-modified sticky rice–lime paste has a denser interior and better macroscopic properties.

## 1. Introduction

In the long history of ancient architecture, lime as the main gelling material of the building mortar has been the most widely used [1,2]. In long-term practice, ancient craftsmen tried to make organic–inorganic composite lime paste by adding various natural organic materials, such as animal blood, plant sap, proteins, oils, sugars, etc. [3], to improve the properties of the lime paste, such as enhancing the strength, regulating the setting time and improving bonding and corrosion resistance [4]. One of them is to add glutinous rice pulp, as the branched chain starch of sticky rice paste can regulate the carbonisation process of lime paste so that the calcium carbonate crystals generated by the carbonisation reaction are finer and denser, thus effectively improving the physical properties, mechanical strength and compatibility of the lime after carbonisation [5,6]. It has been confirmed by many historical sites that it was widely used in ancient masonry buildings [1,2,5,6,7].

At present, in the repair work of ancient masonry buildings, it is necessary to consider the coordination of the performance between the repair material and the old structure [8]. If cement-based restoration materials are used, there are many problems, such as excessive strength, low permeability and precipitation of soluble salts [9]. Studies have shown that the use of traditional materials prevents the weakening of the main body by creating new weak points due to the excessive difference in strength between modern restoration materials and ancient masonry materials [10], considering that lime-based materials have the best compatibility with ancient building materials, and that sticky rice–lime paste, as a traditional ancient building material and easy to obtain, is beginning to gradually replace cement mortar as the mainstream ancient masonry building repair material [6,10]. However, for sticky rice–lime paste in the restoration of ancient buildings, there are some defects, such as longer setting time, low strength, greater shrinkage, poor water resistance and insufficient freeze–thaw resistance [7,11,12]. The research found that various admixtures were added within the lime paste to fill the internal pores of the paste, promote hydration and improve compactness to improve the properties of the lime paste, such as fibres, aluminium sulphate, nanosilica (NS), nano-titanium dioxide and graphene [8,13,14,15]. Therefore, this can provide some references for improving the properties of sticky rice–lime paste.

Graphene oxide (GO) is a derivative of graphene with a high specific surface area and abundant surface functional groups [16]. Studies have shown that GO can improve the mechanical properties of cement-based composites [17,18,19]. Long et al. [20] concluded that GO can promote the hydration of cement, increase the density of calcium silicate hydrate (C–S–H) and refine the pore structure to inhibit the extension of cracks. While there are relatively few studies on the composite of GO with lime-based materials, Faria et al. [13] showed that GO can improve the mechanical properties as well as the water resistance of lime mortar. Although GO has good dispersibility in water, in alkaline environments, divalent cations such as Ca^2+^ cause cross-linking of GO [21], leading to severe agglomeration of GO and affecting its modification effect [22]. To solve this problem, researchers have tried to improve the dispersion of GO in cement by ultrasonication and the addition of surfactants and silica powder [19,23,24]. Nanosilica (NS) has also been less studied in lime or sticky rice–lime, where the addition of NS can exert the filler effect and volcanic ash effect of NS while promoting carbonation, which can lead to improved strength and durability [15,25].

In order to solve the problems of poor dispersion and easy agglomeration of GO in alkaline materials, so that it can further enhance the compactness and improve the durability properties of sticky rice–lime paste, this study prepares graphene oxide–nano-silica hybrids (GO–NS) by attaching nano-silica (NS) to the surface of GO nanosheets through a chemical preparation process. The aim is that the NS particles in the hybrids are able to separate the GO nanosheets and reduce the effect of van der Waals forces between the GO nanosheets, which improves the dispersion of GO and promotes the carbonation by providing a higher number of nucleation sites [26]. In addition, the active NS reacts with Ca(OH)_2_ to produce high-strength C–S–H, and fills the internal pores of the lime-based material to enhance the compactness of the hardened lime-based material [15,27]. It was found that very few studies have been conducted on the composite of GO–NS hybrids with sticky rice–lime paste, so in this study, GO–NS was added into sticky rice–lime paste with different contents to prepare GO–NS-modified sticky rice–lime paste. The effects of GO–NS on the physical properties of sticky rice–lime paste were investigated by three tests: consistency, setting time and shrinkage. The effects of GO–NS on the durability performance of sticky rice–lime paste were investigated by a water resistance test and freeze–thaw cycle resistance test, and the modification mechanism of GO–NS on sticky rice–lime paste was revealed by various characterisation tests. This study can provide a certain reference for the application of GO–NS-modified sticky rice–lime paste in the repair work of ancient buildings.

## 2. Materials and Methods

### 2.1. Raw Materials

The ethanol (C_2_H_5_OH) used was 95% alcohol produced by Shandong Anjie Gaoke Disinfection Technology Co., Ltd. (Dezhou, China). GR-grade ammonia (NH_3_-H_2_O) with a concentration of 25%~28% was produced by Tianjin Komeo Chemical Reagent Co (Tianjin, China). The concentration of 98% AR-grade ethyl orthosilicate (THOS) was produced by Tianjin Damao Chemical Reagent Factory (Tianjin, China). Distilled water was produced by Shandong Leilin Trading Co., Ltd. (Linyi, China). GO used research-grade single-layer graphene oxide produced by Suiheng Technology Company (Shenzhen, China), and the specific technical parameters are shown in Table 1. For lime, calcium hydroxide (analytically pure) was used, with a content of 95% or more. The water-reducing agent was polycarboxylic acid high-efficiency water-reducing agent (PCE) produced by Hunan Zhongyan Building Materials Technology Co., Ltd. (Yueyang, China). Sticky rice flour was produced by Jingshan Jieyu Trading Co., Ltd. (Ningbo, China).

### 2.2. Preparation of GO–NS

Access to GO–NS was achieved by in situ hydrolysis and condensation of ethyl orthosilicate using a sol–gel method to attach NS particles to GO nanosheets [28,29,30,31]. An amount of 300 mL of ethanol was added to a suspension made from 120 mg of GO with 12 mL of distilled water, and the mixture was ultrasonicated in a water bath for 1 h. Then, 5 mL of ammonia was dropped to adjust the pH to about 9 before ultrasonication for 1 h. Next, 3 mL of ethyl orthosilicate was added with the aim of hydrolysing it in the mixture to obtain NS. The mixture was then ultrasonicated for 3 h to obtain a GO–NS dispersion. To ensure complete reaction, the prepared GO–NS dispersion was left at room temperature for 2 days. After centrifugation and washing operations, an impurity-free GO–NS dispersion was obtained. Then, GO–NS powder was obtained after vacuum drying at 60 °C.

**Figure 1:**
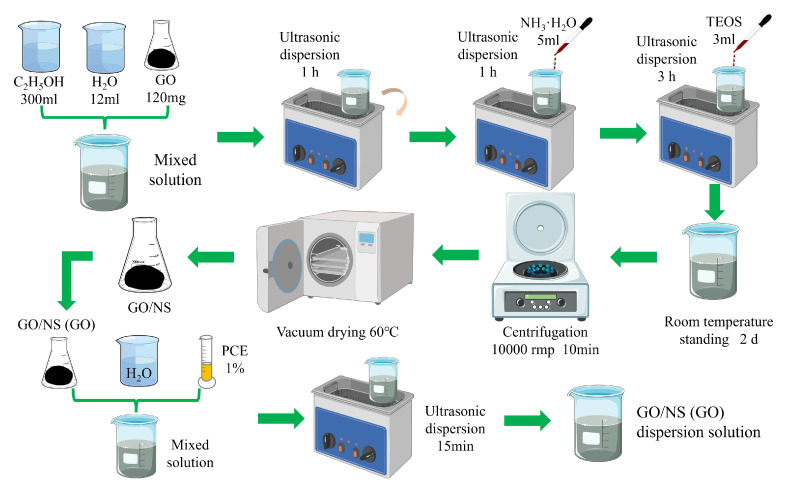
GO–NS (GO) dispersion preparation flowchart.

In order to make GO achieve a better dispersion effect in a lime paste (alkaline) environment, polycarboxylic acid high-efficiency water-reducing agent (PCE) was used as the dispersing agent in this study, and its content was 1% [32,33]. GO and GO–NS were mixed with the corresponding proportions of PCE and water, respectively, ultrasonicated for 15 min and then cooled to room temperature to obtain well-dispersed GO and GO–NS solutions. The specific preparation parameters and process are shown in Figure 1.

The instruments and parameters used in the preparation process are shown in Table 2.

### 2.3. Production of Modified Sticky Rice–Lime Paste Specimens

To analyse the effects of GO and GO–NS on the properties of sticky rice–lime paste, respectively, the contents of GO and GO–NS (mass percent) added to the sticky rice–lime paste were 0.02%, 0.04%, 0.06% and 0.08%. Therefore, each modified sticky rice–lime paste was prepared based on the traditional mixing ratio of sticky rice–lime paste; i.e., the content of sticky rice powder was 5%, and there was a water–lime ratio of 0.8 and 1% PEC. It should be noted that the sticky rice powder was added by boiling it into the form of sticky rice paste, so the water in the sticky rice paste needs to be deducted when adding water. The specific mix ratio design is shown in Table 3.

Referring to the specification JGJ70-2009 [34], sticky rice–lime paste was mixed according to the mixing ratio, and cubic specimens with dimensions of 50 mm × 50 mm × 50 mm and prismatic specimens with dimensions of 40 mm × 40 mm × 160 mm were prepared. All sticky rice–lime paste specimens were kept at 20 ± 2 °C and 60 ± 5% relative humidity for 2 d and then demolded; after which, they were cured under the same conditions until 90 d.

### 2.4. Test Methods

#### 2.4.1. Physical Property Tests

In order to ensure that sticky rice–lime paste has good fluidity, based on JGJ/T 70-2009 [34], The SC-145 mortar consistency tester produced by Lanjian Zhongyi Instrument Equipment Co., Ltd. (Cangzhou, China) was used to determine the consistency of sticky rice–lime paste, which was used to measure the fluidity of modified sticky rice–lime paste. 1. In order to understand the reasonable construction time of sticky rice-lime paste, with reference to GB/T 1346-2011 [35], the initial setting time and final setting time of sticky rice-lime paste were determined using an ISO standard Vicat apparatus produced by Beijing Zhongjiao Jianyi Technology Development Co., Ltd. (Beijing, China). Due to the objective fact that there is shrinkage of lime-based materials, the volume change of sticky rice–lime paste in the hardening process needs to be taken into account, so with reference to the standard JGJ/T 70-2009 [34], a vertical mortar shrinkage meter of type 176 produced by Shaoxing Baojia Instrument Co., Ltd. (Shaoxing, China) was used to determine the length of prismatic specimens at the ages of 7 d, 14 d, 21 d, 28 d, 56 d and 90 d, respectively. Shrinkage heads have been buried at both ends of the specimens in advance. The natural drying shrinkage rate of the specimens can be calculated using Equation (1).

(1) $$\varepsilon_{\mathrm{at}}=\frac{L_{0}-L_{t}}{L-L_{d}}$$

where *ε_at_* is the natural drying shrinkage rate (%) at the corresponding age; *L*_0_ is the length of 7 d, i.e., initial length (mm); *L_t_* is the measured length of the specimen at the corresponding age (mm); *L* is the length of the specimen, taken as 160 mm; *L_d_* is the sum of the length of the two shrinkage heads buried in the lime paste, i.e., 20 ± 2 mm.

#### 2.4.2. Durability Test

The durability testing of sticky rice–lime paste included both water resistance and freeze–thaw cycles. The water resistance test referred to the specification JGJ/T 70-2009 [34] using cubic specimens, which were weighed after drying at a temperature of 78 ± 3 °C for 48 ± 0.5 h. Then, the specimens were placed in a constant-temperature water bath where the surface of the specimen was about 20 mm from the water’s surface, the water temperature was 20 ± 2 °C and the immersion time was 24 h and 48 h. After immersion, the specimen was removed and weighed to calculate its water absorption rate using Equation (2). The cubic compressive strength of the specimen before and after immersion in water was tested using a WDW-100 universal testing machine manufactured by Changchun Xinke Testing Equipment Co., Ltd. (Changchun, China) . The loading rate was 5 mm/min, and the results were calculated using Equation (3). The compressive strength of sticky rice–lime paste before and after water absorption was compared, and finally, the softening factor was calculated to evaluate the water resistance of sticky rice–lime paste, which was calculated using Equation (4). The larger the softening coefficient, the smaller the loss of strength of the lime paste after immersion and the better its water resistance.

(2) $$W=\frac{m_{1}-m_{0}}{m_{0}}$$

where *W* is the water absorption rate of the specimen (%); *m*_1_ is the mass of the specimen after water absorption (g); *m*_0_ is the mass of the dry specimen (g).

(3) $$f_{0}=\frac{N_{u}}{A}$$

where *f*_0_ is the compressive strength of cubic specimen (MPa); *N*_u_ is the failure load of the specimen (N); *A* is the pressure-bearing area of the specimen (mm^2^).

(4) $$K=\frac{f_{n}}{f_{0}}$$

where *K* is the softening factor; *f*_n_ is the compressive strength of the specimen after software (MPa); *f*_0_ is the compressive strength of the specimen of the unsoftened part (MPa).

The freeze–thaw cycle test was based on JGJ/T70-2009 [34]. Cubic specimens were immersed in water 2 d before 90 d of age, and freeze–thaw cycles started at 90 d. A freeze–thaw cycle process consists of freezing in a refrigerator at −20 °C for 4 h and thawing in water at 20 °C for 4 h. Failure of the freeze–thaw cycle was determined by the specimens showing severe cracking, peeling of the skin and missing blocks. The maximum number of freeze–thaw cycles that sticky rice–lime paste can withstand is recorded as the freeze–thaw cycle life. The morphological damage, mass loss and strength loss of the specimens were used to jointly evaluate their freeze–thaw resistance.

After freeze–thaw cycles, the specimens were dried to a constant weight in an oven at 60 °C. The cubic compressive strength was then measured and the rate of strength loss of lime paste was calculated from Equation (5). The specimens were weighed after every five cycles completed. and the rate of specimen mass loss was calculated according to Equation (6).

(5) $$\Delta F=\frac{f_{0}-f_{n}}{f_{0}}$$

where ΔF is the strength loss rate (%) of the specimen after freeze–thaw cycle; *f_0_* is the compressive strength value (MPa) of the specimen without freeze–thaw cycle; *f*_n_ is the compressive strength value (MPa) of the specimen after the nth freeze–thaw cycle.

(6) $$\Delta W=\frac{m_{0}-m_{n}}{m_{0}}$$

where Δ*W* is the mass loss rate (%) of the specimen after freeze–thaw cycle; *m*_0_ is the mass (g) of the specimen without freeze–thaw cycle; *m*_n_ is the mass (g) of the specimen after the nth freeze–thaw cycle.

#### 2.4.3. Characterisation Technique

The characterisation technique is divided into two main parts. In the first part, to prove the successful preparation of GO–NS hybrids, Phenom XL G2 scanning electron microscope (SEM)manufactured by Phenom Scientific Instruments (Shanghai) Co., Ltd. (Shanghai, China) and FEI F20 field emission transmission electron microscope (TEM) manufactured by FEI Company (Hillsboro, OR, USA) were used to observe the microstructure and surface morphology of GO–NS. A MiniFlex 600 X-ray diffractometer (XRD) manufactured by Rigaku Holdings Corporation (Tokyo, Japan) was used to analyse the structural characteristic peaks of GO–NS with a scanning range of 5° to 60° (2θ), a step size of 0.02° (2θ) and a scanning rate of 2°/min. A Nicolet iS50 Fourier transform infrared spectrometer (FTIR) manufactured by Thermo Fisher Scientific (Waltham, MA, USA) was used to analyse the characteristic functional groups of GO–NS, with a scanning range of 400–4000 cm^−1^, a resolution of 4 cm^−1^ and 32 scans.

In the second part, to characterise the products and microstructure of GO–NS-modified sticky rice–lime paste, XRD and FTIR were used to analyse the physical phase of the modified sticky rice–lime paste using the same methods as in the first part, and the surface morphology of the sticky rice–lime paste was observed using a JSM-7800F thermal field emission scanning electron microscope (FESEM) manufactured by Japan Electron Optics Laboratory Co., Ltd. (Tokyo, Japan).

## 3. Results and Discussions

### 3.1. Characterisation of GO–NS

**Figure 2:**
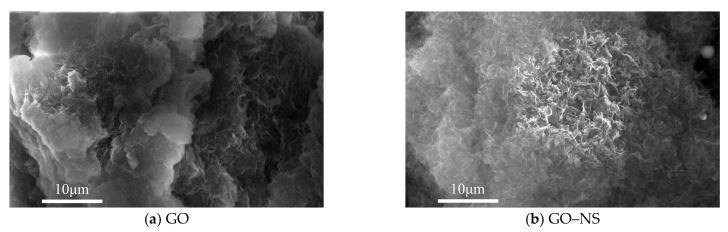
SEM of GO and GO–NS.

Figure 2 shows the SEM results of GO and GO–NS. It can be observed that GO (Figure 2a) sheets are stacked between each other due to van der Waals forces, and a clear agglomeration phenomenon is formed. Figure 2b shows that the GO–NS sheets are relatively dispersed from each other and do not show obvious agglomerates. The reason is that a large number of NS particles are attached to the surface of the hybridised GO, resulting in isolation, weakening the van der Waals forces between the GO sheets and reducing the stacking between the GO–NS sheets.

**Figure 3:**
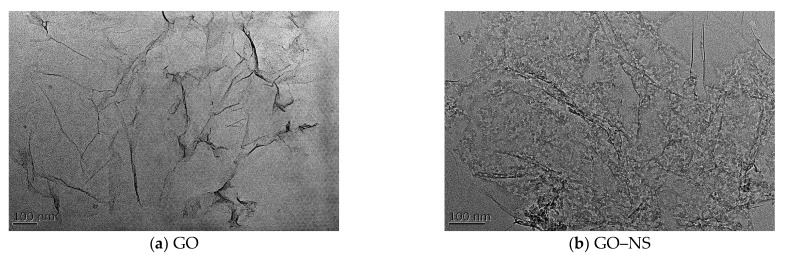
TEM of GO and GO–NS.

Figure 3 shows the TEM results of GO and GO–NS. It can be observed that the hybridised GO–NS maintains the initial sheet structure of GO while the NS particles are densely adhered to the GO sheet, and the spherical particles of about 5–20 nm can be clearly observed to be uniformly adhered to the interlayer of GO.

**Figure 4:**
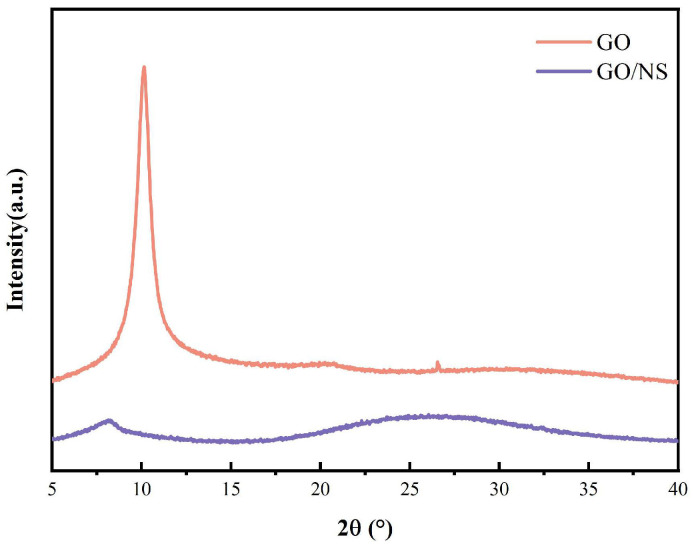
XRD of GO and GO–NS.

Figure 4 shows the XRD results of GO and GO–NS. It can be seen that there is a sharp peak of GO at 2θ = 10.14°, which is the characteristic peak of GO [26,36]. In contrast, the GO characteristic peak in the GO/NS X-ray diffraction results was decreased, showing a typical broad peak caused by amorphous NS at around 2θ = 25° [26,36]. This is due to the fact that NS is tightly attached to the GO sheet layer to change the characteristic structure of GO, resulting in the disappearance of the characteristic peaks of GO and the appearance of the characteristic peaks caused by NS.

**Figure 5:**
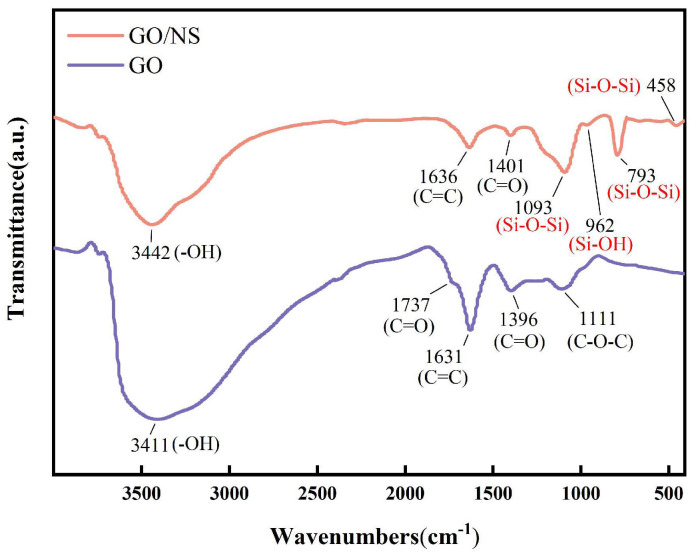
FTIR of GO and GO–NS.

Figure 5 shows the FTIR results of GO and GO–NS, and it can be observed that four new absorption peaks have been added to GO–NS. The absorption peaks at 1093 cm^−1^, 793 cm^−1^ and 458 cm^−1^ are bending vibrations of the Si–O–Si functional group, while the absorption peak near 962 cm^−1^ corresponds to the stretching vibration of the Si–OH functional group, and all of these peaks proved the existence of the NS structure [36].

Combining the results of the four microscopic tests of SEM, TEM, XRD and FTIR, it can be shown that the NS particles are uniformly attached to the surface of the GO sheet layer, and the GO–NS hybrids are successfully prepared.

### 3.2. Physical Property Analysis

#### 3.2.1. Consistency

**Figure 6:**
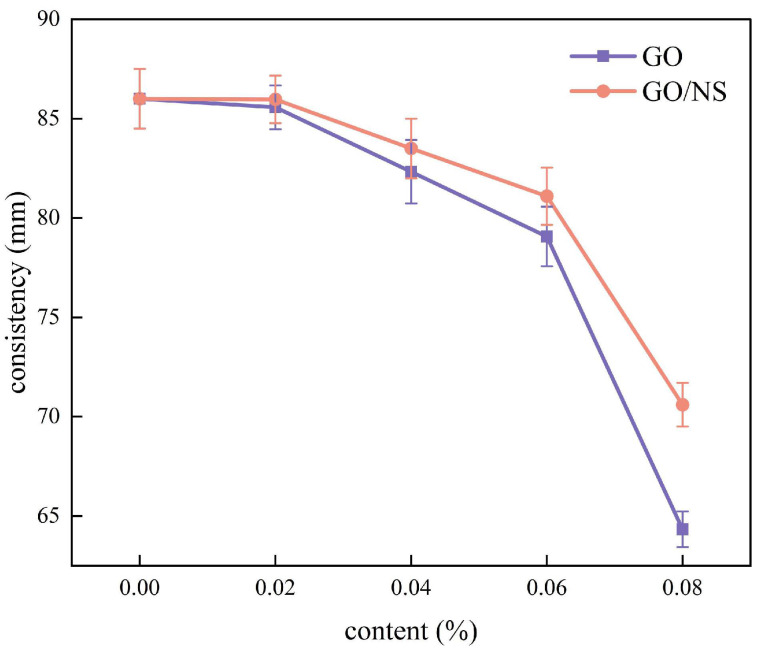
Consistency values of GO and GO–NS-modified sticky rice–lime paste.

The variation of the consistency of modified sticky rice–lime paste with GO and GO–NS content is shown in Figure 6. It can be seen that the effect of GO and GO–NS on the consistency is not significant when the content is small (less than 0.02%), but as the content increases (0.02–0.08%), the consistency gradually decreases. A comparison of GO and GO–NS shows that the decreases in the consistency of GO–NS are smaller than that of GO, indicating that the effect of GO–NS on the fluidity of sticky rice–lime paste is smaller than that of GO.

#### 3.2.2. Setting Time

**Figure 7:**
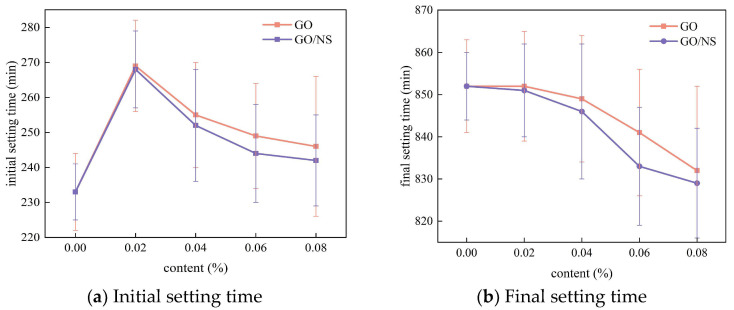
Setting time of GO and GO–NS sticky rice–lime paste.

The setting time of each sticky rice–lime paste is shown in Figure 7. For the initial setting time, both GO and GO–NS significantly increased the initial setting time of sticky rice–lime paste at low content (0.02%), and the initial setting time gradually decreased with the increase of the content, but all of them were greater than that of pure sticky rice–lime paste. For the final setting time, GO and GO–NS did not have much effect on the final setting time at the content of 0.02%, while the final setting time gradually decreased with the increase of the content. It is shown that the appropriate content of GO and GO–NS can increase the initial setting time and shorten the final setting time, which makes the masonry structure of ancient buildings in the reinforcement repair work. Compared with the pure sticky rice–lime paste, the modified sticky rice–lime paste has more working time before the initial setting, and it can reach the final state to play the bonding effect earlier, which is favourable to the reinforcement repair work. Apparently, with a content of 0.02%, GO–NS is slightly superior to GO in its ability to increase the initial setting time and shorten the final setting time.

#### 3.2.3. Shrinkage Rate

**Figure 8:**
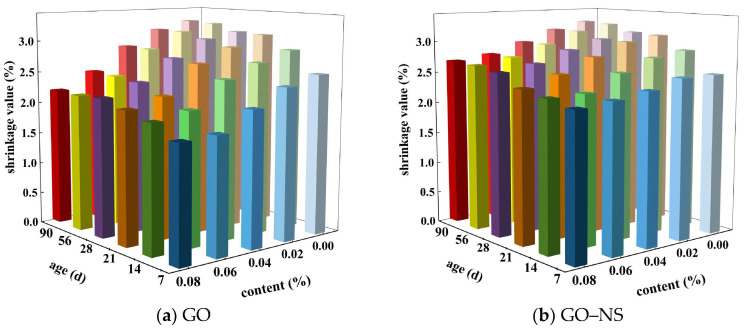
Shrinkage rate of GO and GO–NS-modified sticky rice–lime paste.

**Figure 9:**
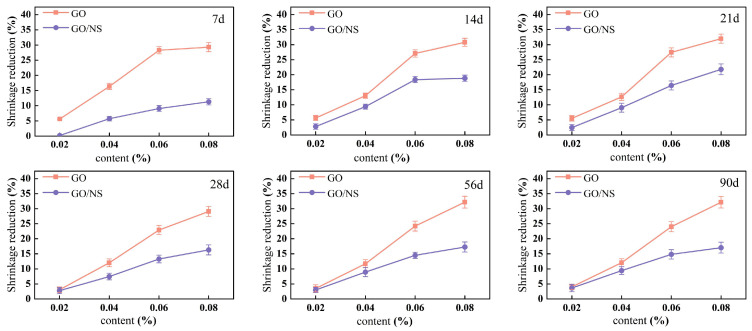
Shrinkage reduction at various ages.

The test results for each age during the natural drying shrinkage test are shown in Figure 8. It can be seen that the natural drying shrinkage of both GO and GO–NS-modified sticky rice–lime paste increased gradually with the increase of age, and the increase was larger in the early stage and stabilised in the late stage. The shrinkage reduction of GO and GO–NS-modified sticky rice–lime paste compared to pure sticky rice–lime paste was analysed according to Figure 8, as shown in Figure 9. It can be seen that the inhibitory effects of both GO and GO–NS on the shrinkage of sticky rice–lime paste at different ages show a tendency to increase with the increase of content. These indicate that the addition of GO and GO–NS can effectively inhibit the shrinkage of sticky rice–lime paste, but the inhibition effect of GO–NS is weaker than that of GO at all ages.

### 3.3. Durability Analysis

#### 3.3.1. Water Resistance

**Figure 10:**
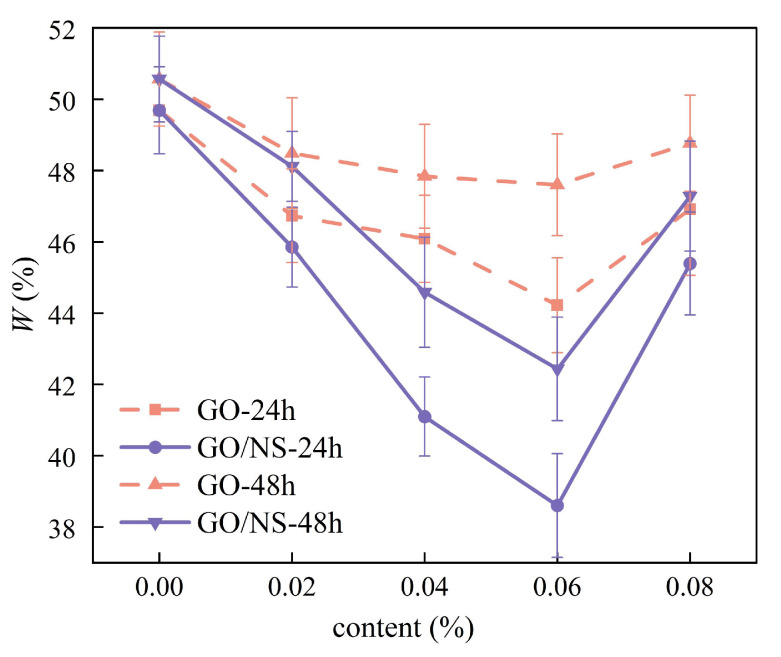
Curves of water absorption versus content.

The water absorption of modified sticky rice–lime pastes after 24 h and 48 h of water immersion is shown in Figure 10. It can be seen that the water absorption of pure sticky rice–lime paste at 48 h did not increase significantly compared with that at 24 h, and both were around 50%, which indicated that the pure sticky rice–lime paste was close to a water-saturated state after 24 h of immersion. The water absorption of both GO and GO–NS-modified sticky rice–lime paste was smaller than that of pure sticky rice–lime paste, and their 24 h water absorption was significantly smaller than that of 48 h, indicating that 24 h of water immersion could not make them close to a water-saturated state, and the addition of GO and GO–NS could effectively inhibit the entry of water. Regardless of the water immersion time, the water absorption of GO–NS-modified sticky rice–lime paste was smaller than that of GO-modified sticky rice–lime paste, which indicated that the water inhibition effect of GO–NS was better than that of GO. The water absorption of both GO and GO–NS-modified sticky rice–lime paste showed a decreasing and then increasing trend with increasing content, with a turning point of 0.06%; i.e., the best water resistance was achieved at this content.

**Figure 11:**
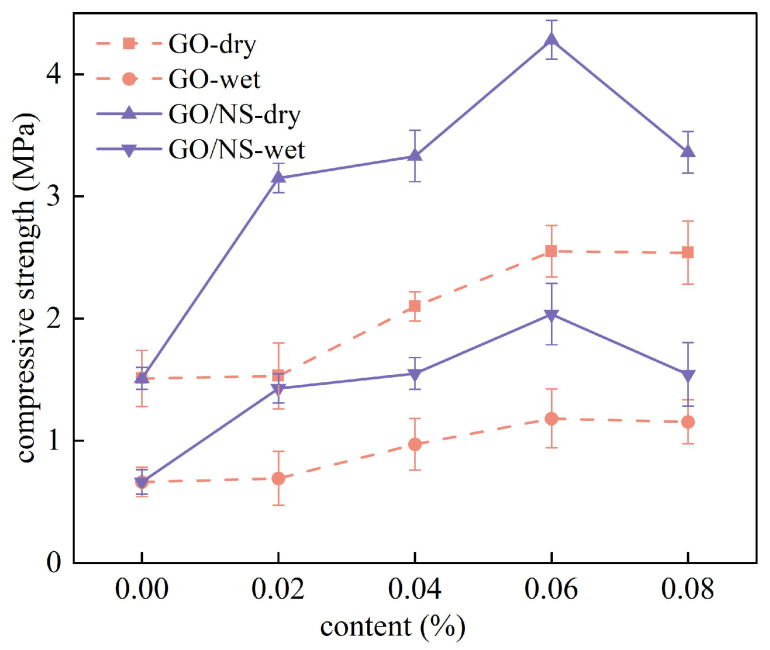
Comparison of strength in wet and dry states.

The cubic compressive strength of the dried and immersed (48 h) modified sticky rice–lime paste is shown in Figure 11. It can be seen that the compressive strengths of both GO and GO–NS-modified sticky rice–lime paste were significantly higher than that of pure sticky rice–lime paste in both dry and wet states, indicating that the addition of GO and GO–NS can improve the compressive properties of sticky rice–lime paste. A comparison of the compressive strengths of GO and GO–NS-modified sticky rice–lime paste shows that the compressive strengths of GO–NS-modified sticky rice–lime paste are all significantly greater than those of GO-modified sticky rice–lime paste. And with the increase of GO and GO–NS-content, the strength changes in both dry and wet states showed a trend of increasing and then decreasing, with a turning point of 0.06%, which means that the optimal strength enhancement was reached at this point. Relative to pure sticky rice–lime paste, when the content was 0.06%, the strength of GO in a dry state was enhanced by 68.9% and its strength in a water-immersed state was enhanced by 78.3%. Meanwhile, the strength of GO–NS was enhanced by 183.4% in a dry state and 207.2% in a water-immersed state. This indicates that the strength enhancement of GO–NS for sticky rice–lime paste is significantly stronger than that of GO.

**Figure 12:**
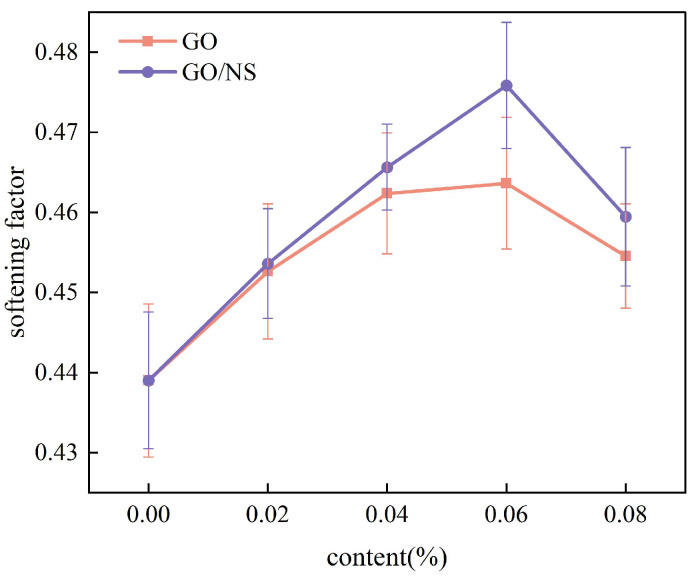
Softening factor.

The softening factor of each sticky rice–lime paste was calculated according to Figure 11, as shown in Figure 12. It can be seen that the softening factors of both GO and GO–NS-modified sticky rice–lime paste are greater than that of pure sticky rice–lime paste, indicating that both can improve the strength loss of pure sticky rice–lime paste due to the effect of water. With the increase of GO and GO–NS dosage, the softening coefficients show both a tendency to increase and then decrease, with a turning point of 0.06%, which means the best resistance to strength loss. Obviously, the softening factor of GO–NS-modified sticky rice–lime paste at this content is much larger than that of GO-modified sticky rice–lime paste, indicating that the inhibition effect of GO–NS on the strength loss of sticky rice–lime paste due to the effect of water is stronger than that of GO.

#### 3.3.2. Freeze–Thaw Cycle Resistance

(1) Mass loss

The maximum number of freeze–thaw cycles that each sticky rice–lime paste specimen can withstand and the typical damage patterns are given in Table 4. It can be seen that both GO and GO–NS prolonged the freeze–thaw cycle life of pure sticky rice–lime paste, and with the increase of the content, it showed a tendency of increasing and then decreasing. The turning point was 0.06%, and obviously the number of freeze–thaw cycles of the GO–NS-modified sticky rice–lime paste was greater than that of the GO-modified sticky rice–lime paste.

**Figure 13:**
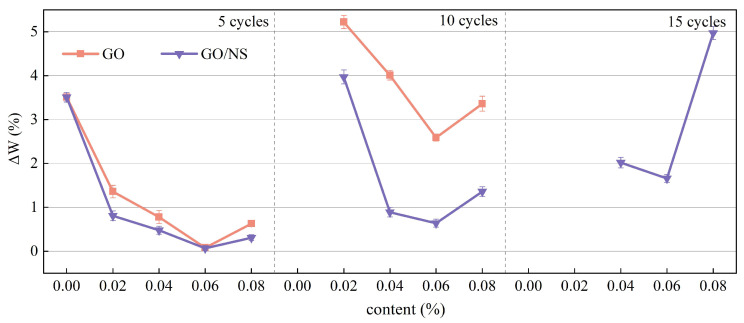
Mass loss rate under freeze–thaw cycle.

The mass loss rate of each sticky rice–lime paste specimen under different numbers of freeze–thaw cycles is shown in Figure 13. It can be seen that after five freeze–thaw cycles, the mass loss of both GO and GO–NS-modified sticky rice–lime paste is smaller than that of pure sticky rice–lime paste, but there is no significant difference between them. However, after 10 freeze–thaw cycles, the pure sticky rice–lime paste had failed, and the mass loss of GO–NS-modified sticky rice–lime paste was significantly lower than that of GO-modified sticky rice–lime paste. After reaching 15 freeze–thaw cycles, the GO-modified sticky rice–lime paste had failed completely, and only three groups of 0.04%, 0.06% and 0.08% of the GO–NS-modified sticky rice–lime paste remained. Overall, the mass loss showed a decreasing and then increasing trend with the increase of GO and GO–NS content—both having reached the lowest mass loss at 0.06%—and an increasing trend with the increase of the number of freeze–thaw cycles. These illustrate that both GO and GO–NS improved the freeze–thaw resistance of sticky rice–lime paste to a certain extent, and the appropriate content can achieve the best improvement effect. The ability of GO–NS to improve the freeze–thaw resistance is better than that of GO.

(2) Strength loss

**Figure 14:**
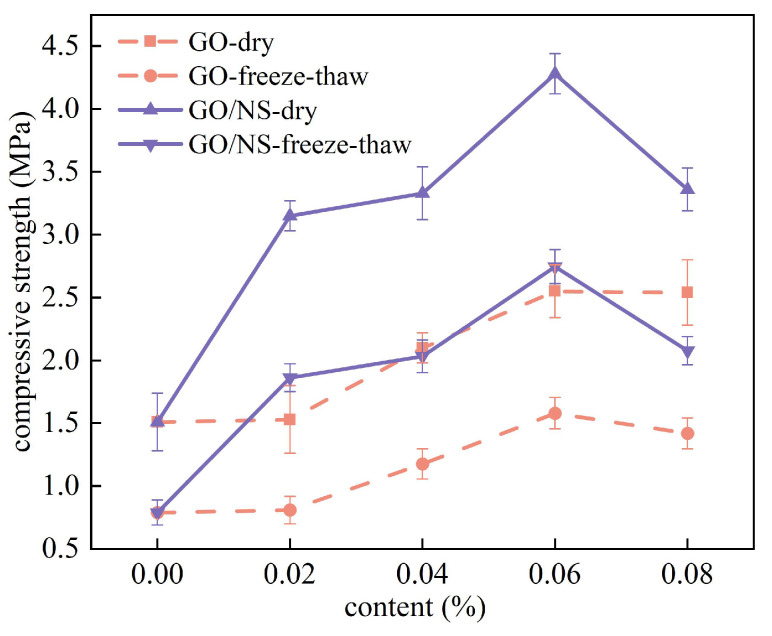
Cubic compressive strength after freeze–thaw cycles.

After the freeze–thaw cycle of the specimen, under a compressive strength test, all the specimens must ensure a good appearance and integrity. For this reason, after observation, the specimens after two freeze–thaw cycles were selected to carry out a cubic compressive strength test, and the results are shown in Figure 14.

As can be seen in Figure 14, the cubic compressive strength of both GO and GO–NS-modified sticky rice–lime paste decreased to different degrees after two freeze–thaw cycles, and the trend of compressive strength change coincided with that of the unfrozen–thaw cycle. The compressive strength was optimised when both GO and GO–NS content reached 0.06%. At a content of 0.06%, the strength of GO-modified sticky rice–lime paste increased by 99.9% over pure sticky rice–lime paste after freeze–thaw cycles, while GO–NS-modified sticky rice–lime paste increased by 247.6%.

**Figure 15:**
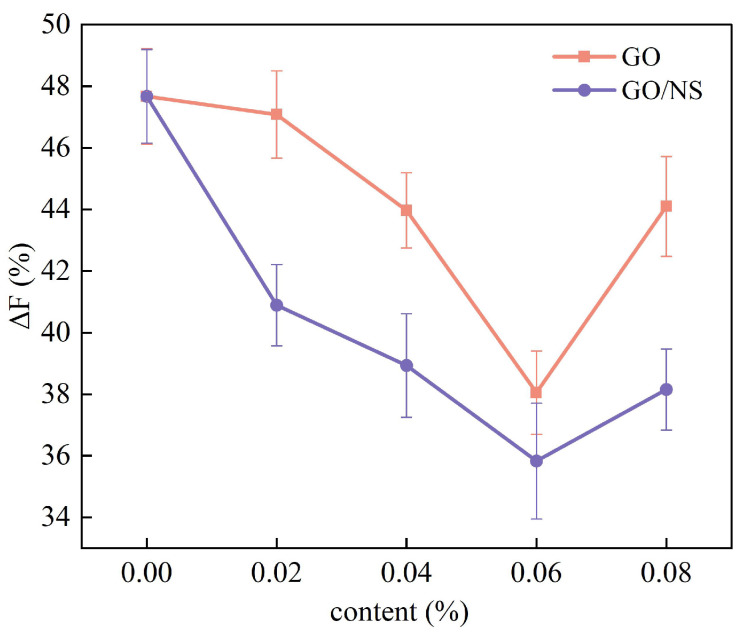
Strength loss rate.

Figure 15 shows the strength loss rate after freeze–thaw cycles. It can be observed that the strength loss rate of modified sticky rice–lime paste shows a decreasing and then increasing trend with the increase of GO and GO–NS content. It was also shown that at 0.06% content, GO and GO–NS-modified sticky rice–lime paste had the lowest strength loss rate: 38.1% for GO and 35.8% for GO–NS. Therefore, based on the strength increase and strength loss rate after freeze–thaw, both GO and GO–NS were able to improve the freeze–thaw resistance of sticky rice–lime paste, and GO–NS was superior compared to GO, which was consistent with the mass loss analysis.

### 3.4. Characterisation of GO–NS-Modified Sticky Rice–Lime Paste

Since the best modification effect was achieved at 0.06% for both GO and GO–NS, the characterisations were carried out with modified sticky rice–lime paste with 0.06% content of the modifier.

#### 3.4.1. XRD and FTIR

**Figure 16:**
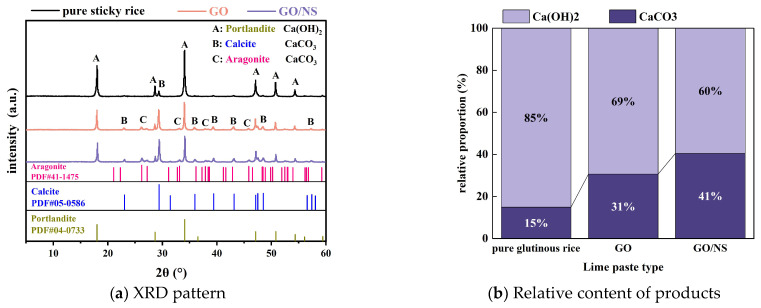
XRD analysis results of each sticky rice–lime paste.

Figure 16 shows the XRD results of each sticky rice–lime paste after setting and hardening. The main hardening products of each sticky rice–lime paste are calcium carbonate (CaCO_3_) and calcium hydroxide (Ca(OH)_2_), as shown in Figure 16a, in which CaCO_3_ was detected as calcite crystals in the pure sticky rice–lime paste, whereas the calcium carbonate in the GO and GO–NS-modified sticky rice–lime pastes embodied calcite and aragonite crystals. It can be seen that the number of diffraction peaks of CaCO_3_ in GO and GO–NS-modified sticky rice–lime paste increased significantly relative to that of pure sticky rice–lime paste, the intensity of CaCO_3_ diffraction peaks at 2θ = 29° increased significantly, and the intensity of Ca(OH)_2_ diffraction peaks at 2θ = 18° and 2θ = 34° decreased significantly. The results of the semi-quantitative analysis are shown in Figure 16b, where the relative content of CaCO_3_ in each sticky rice–lime paste is ranked as follows: pure sticky rice < GO < GO–NS, and the relative content of Ca(OH)_2_ is correspondingly reduced. These results show that GO with GO–NS promotes the carbonation reaction of lime, consuming a part of Ca(OH)_2_; the reaction is shown in Equation (7).

(7) $$\mathrm{Ca}{\left(\mathrm{OH}\right)}_{2}+CO_{2}+H_{2}O\to \mathrm{Ca}{\mathrm{CO}}_{3}+\left(n+1\right)H_{2}O$$

(8) $$Ca{\left(\mathrm{OH}\right)}_{2}+\mathrm{Si}O_{2}+H_{2}O\to \mathrm{CaO}\cdot {\mathrm{SiO}}_{2}\cdot H_{2}O$$

**Figure 17:**
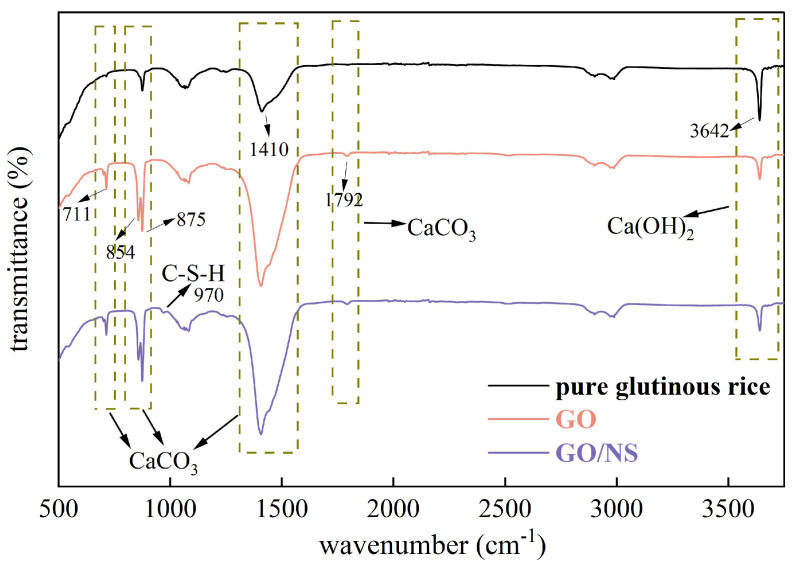
FTIR spectra of each sticky rice–lime paste.

Figure 17 shows the FTIR spectra of each of the sticky rice–lime pastes. Absorption peaks at approximately 711 (in-plane bending vibration), 875 (out-of-plane bending vibration), 1410 (antisymmetric telescoping vibration) and 1792 (stretching vibration) cm^−1^ induced by ${\mathrm{CO}}_{3}^{2-}$ in calcite (CaCO_3_) can be observed [12,37,38], as well as aragonite (CaCO_3_) absorption peak at approximately 854 cm^−1^ (out-of-plane bending vibration) [12,39]. The absorption peak induced by O–H in Ca(OH)_2_ is observed at approximately 3642 cm^−1^ (stretching vibration) [37,38,40], and an absorption peak can be seen at approximately 970 cm^−1^ (asymmetric stretching vibration) in C–S–H induced by Si–O [38,40,41]. The comparison shows that the absorption peaks at 711, 875, 1410 and 1792 cm^−1^ of GO and GO–NS-modified sticky rice–lime paste are significantly enhanced compared to pure sticky rice–lime paste, indicating an increase in the hardening product calcite (CaCO_3_). Further, the pure sticky rice–lime paste does not show an absorption peak at 854 cm^−1^, indicating that GO and GO–NS-modified sticky rice–lime paste additionally generates aragonite (CaCO_3_). The absorption peaks at 3642 cm^-1^ of GO and GO–NS-modified sticky rice–lime paste were significantly weaker than that of pure sticky rice–lime paste, indicating less Ca(OH)_2_ content. It is noteworthy that only the GO–NS-modified sticky rice–lime paste has a small absorption peak at 970 cm^−1^, indicating the presence of C–S–H in the product, and a small portion of Ca(OH)_2_ in the GO–NS-modified sticky rice–lime paste is consumed in the process; the reaction formula is shown in Equation (8).

Thus, XRD together with FTIR demonstrated that GO and GO–NS contributed to the generation of more CaCO_3_ from the sticky rice–lime paste, as well as the additional generation of C–S–H due to the presence of NS.

#### 3.4.2. SEM

(1) Microstructure of hardened sticky rice–lime paste

**Figure 18:**
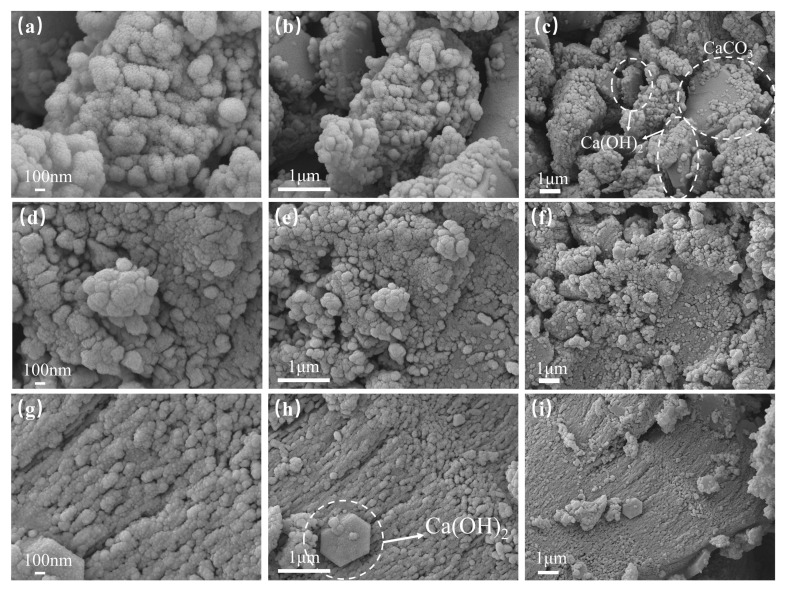
Microscopic morphology of each sticky rice–lime paste. (**a**–**c**) pure sticky rice–lime paste; (**d**–**f**) GO-modified sticky rice–lime paste; (**g**–**i**) GO–NS-modified sticky rice–lime paste.

Due to the presence of sticky rice, it will have an effect on the microscopic morphology of the hardening product of the sticky rice–lime paste, forming a kind of organic–inorganic composite structure, and it is difficult to directly observe the original crystal morphology [2,37]. For this reason, the pore structure of the sticky rice–lime paste is mainly analysed here. Figure 18 shows the microscopic morphology of each of the sticky rice–lime pastes in the same area and at different magnifications. It can be seen that the surface of each sticky rice–lime paste exhibits a rounded, granular texture due to the effect of the sticky rice. As the magnification decreases, it can be seen that the microstructure of the pure sticky rice–lime paste is very loose (Figure 18a–c). There are more pores and crevices, and hexagonal flaky Ca(OH)_2_ and irregular calcite crystals can be easily observed. In contrast, the microstructures of GO (Figure 18d–f) and GO–NS (Figure 18g–i)-modified sticky rice–lime pastes are more compact, especially GO–NS-modified sticky rice–lime pastes, which are much more compact than that of GO. It is illustrated that the sheet structure of GO and GO–NS provides more nucleation sites and makes the microstructure more compact, while GO–NS improves the disadvantage of GO being prone to agglomeration, so its microscopic pore is significantly reduced and the structure is more compact. Thus, it exhibits a significant improvement of the physical and mechanical properties on the macroscopic level.

(2) Microstructure after freeze–thaw cycles

**Figure 19:**
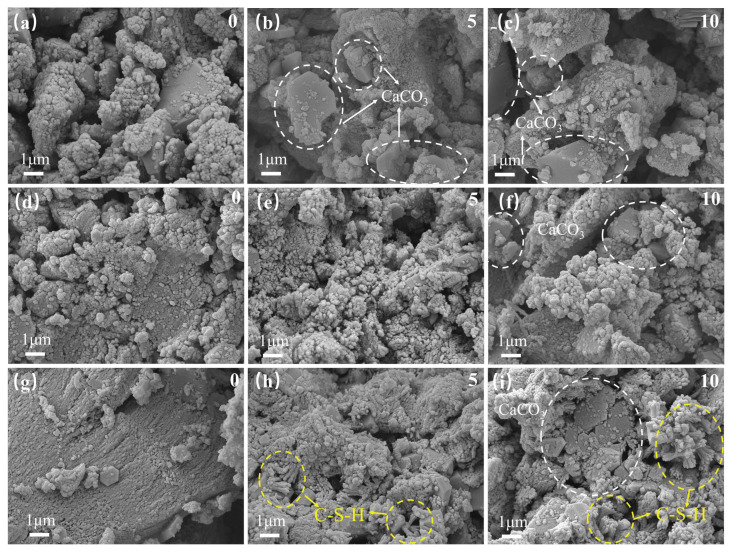
Microscopic morphology of each sticky rice–lime paste after freeze–thaw cycles. (**a**–**c**) pure sticky rice–lime paste; (**d**–**f**) GO-modified sticky rice–lime paste; (**g**–**i**) GO–NS-modified sticky rice–lime paste.

Figure 19 shows the microscopic morphology of each sticky rice–lime paste after undergoing 0, 5 and 10 freeze–thaw cycles at the same magnification. Figure 19a–c shows pure sticky rice–lime paste, Figure 19d–f shows GO-modified sticky rice–lime paste and Figure 19g–i shows GO–NS-modified sticky rice–lime paste. It can be observed that each sticky rice–lime paste shows the phenomenon of pore structure deterioration with the increase of the number of freeze–thaw cycles, which is due to the dissolution of the sticky rice paste in contact with water, as well as the collapse, dissolution and expansion of slaked lime (Ca(OH)_2_) in contact with water. However, compared to pure sticky rice–lime paste, the pore structure of GO and GO–NS-modified sticky rice–lime paste after being subjected to freeze–thaw cycles was improved, and the number of pores, the degree of crevice connectivity and the width and depth of defects were reduced. It can be seen that after being subjected to freeze–thaw, with the dissolution of sticky rice and slaked lime, the characteristics of a hardened product of lime paste are revealed, exposing calcite (aragonite) crystals. In both GO and GO–NS-modified sticky rice–lime pastes, these hardening products were observed to fill in the pores, resulting in a reduction of the pore structure. Further, fibrous C–S–H was observed in the freeze–thawed GO–NS-modified sticky rice–lime paste, which likewise acted as a filler for the pores. For this reason, the GO–NS-modified sticky rice–lime paste was better compacted than the GO-modified sticky rice–lime paste.

### 3.5. The Relationship Between Macro and Micro

In order to explain the mechanism of action of GO and GO–NS on sticky rice–lime paste, the micro-analysis of the two parts of 3.1 and 3.4 was linked to each macro phenomenon. The consistency of modified sticky rice–lime paste showed a decreasing trend with the increase of GO and GO–NS content, which was attributed to the fact that both GO and GO–NS were two-dimensional folded structures with more hydrophilic functional groups on their surfaces [16,42], which had a certain effect of adsorption of water, which would reduce the content of free water in the paste, resulting in a decrease in the fluidity. The more separated layer structure of GO–NS reduces agglomeration, results in a larger specific surface area, exposes more hydrophilic groups, binds more free water and further reduces fluidity. So, the decrease in the fluidity of GO–NS-modified sticky rice–lime paste is significantly smaller than that of GO-modified sticky rice–lime paste.

Similarly, due to the adsorption of water by GO and GO–NS, more water was stored, delaying the initial setting time, but due to the increase in content, the sticky rice–lime paste was thickened and therefore showed a decrease in the initial setting measurement. For the final setting time, GO and GO–NS exhibited a shorter final setting due to their hardening-promoting effect, whereas NS produced additional hydration. For this reason, the final setting time of GO–NS-modified sticky rice–lime paste was slightly shorter than that of GO.

Due to the templating effect of GO and GO–NS, which provide abundant nucleation sites through their own high aspect ratios, the hardening process can be facilitated, while the better interlayer structure of GO–NS can provide more nucleation sites; therefore, this also promotes the carbonation reaction during the hardening process, resulting in a higher amount of CaCO_3_ in the hardened GO–NS-modified sticky rice–lime paste. Its hardening product fills the pores, allowing for less space for shrinkage by drying action, and exhibits the effect of inhibiting the shrinkage of sticky rice–lime paste. Meanwhile, NS with volcanic ash activity in GO–NS reacts with Ca(OH)_2_ in lime paste to produce C–S–H, which produces some chemical shrinkage, as well as drying shrinkage caused by the consumption of more water during the reaction process [15,43]. Thus, GO–NS inhibits the shrinkage weakly under the effect of double shrinkage.

The sheet-layer structure of GO and GO–NS not only plays a bridging effect and mechanical interlocking role inside the paste [26] but also improves the structure of the hardening product of sticky rice–lime paste and makes its internal structure more compact so that it enhances the compressive strength of the sticky rice–lime paste and prevents the infiltration of water, which improves the water-resistance performance and freeze–thaw resistance. However, it is possible that agglomeration occurred due to the high content of GO and GO–NS; i.e., a negative trend was observed above 0.06%. The hydration product C–S–H of GO–NS-modified sticky rice–lime paste is not only stronger but also plays the role of filling, which will not collapse and expand when it meets water, making the structure more compact and showing better compressive strength, water resistance and freeze–thaw resistance.

## 4. Conclusions

In this paper, GO–NS hybrids were prepared with the aim of improving the durability of sticky rice–lime paste, an ancient architectural gelling material. Its effect on the basic physical properties and durability of sticky rice–lime paste was investigated, and the pure sticky rice–lime paste, GO and GO–NS-modified sticky rice–lime paste were studied comparatively. The following conclusions were drawn:

(1). The characterisation of the surface morphology and chemical structure of GO–NS hybrids shows that NS can be successfully attached to the surface of GO, which can increase the interlayer spacing of GO to reduce the stacking effect caused by interlayer van der Waals forces and ultimately give full play to the template role of GO.
(2). GO–NS reduces the fluidity of the sticky rice–lime paste, and is able to significantly prolong the initial setting time and shorten the final setting time. It also reduces the shrinkage of the sticky rice–lime paste, but its ability to reduce shrinkage is slightly weaker than that of GO due to the chemical shrinkage caused by C–S–H in the hardening product of GO–NS.
(3). GO–NS significantly improves the compressive strength of sticky rice–lime paste and reduces water absorption as well as the strength loss after water absorption. Moreover, it can increase the number of freeze–thaw cycles and reduce the mass loss and strength loss after freeze–thaw cycles. When the optimum content of GO–NS is 0.06%, the durability of sticky rice–lime paste can be significantly improved.
(4). The characterisation analysis revealed that GO–NS provided more nucleation sites for the hardening of sticky rice–lime paste due to its superior templating role. GO–NS is able to induce more Ca(OH)_2_ to produce more CaCO_3_, and at the same time, is able to generate C–S–H due to the volcanic ash effect of NS. After the freeze–thaw cycle, with the dissolution of the sticky rice paste and Ca(OH)_2_, the CaCO_3_ crystal structure and the unique C–S–H structure of GO–NS can be observed in the micro-morphology. Therefore, with the synergy of the dual products, the hardened GO–NS-modified sticky rice–lime paste obtained higher compactness and showed better durability.

## Figures and Tables

**Table 1:** Technical parameters of graphene oxide.

Appearances	Layer Diameter/um	Purity/%	Carbon Content/%	Oxygen Content/%	Sulphur Content/%
Brown powder	0.2~5.0	>95	<45	>54	<1

**Table 2:** Instruments required for GO–NS preparation.

Instruments	Type	Parameters	Manufacturer
Ultrasonic disperser	F-100SD	Frequency: 40 KHz, power: 600 W	Shenzhen Fuyang Technology Group Co., Ltd. (Shenzhen, China)
High-speed centrifuge	TG16	Maximum RPM: 16,000 r/min Maximum Relative Centrifugal Force: 17,800× *g*	Hunan Changsha Yingtai Instrument Co., Ltd. (Changsha, China)
Vacuum drying oven	DZF6020	Vacuum: 133 Pa, temperature control: RT + 10~200 °C	Tianjin Zhongjia Instrument Co., Ltd. (Tianjin, China)
Precision electronic balance	CN-LQC1003	Precision: 0.001 g	Kunshan Youkewei Electronic Technology Co., Ltd. (Kunshan, China)

**Table 3:** Sticky rice–lime paste mix ratio design.

Paste Type	Sticky Rice Content/%	Water–Lime Ratio	PEC/%	GO Content/%	GO–NS Content/%
Pure sticky rice–lime paste	5	0.8	1	—	—
GO-0.02				0.02	—
GO-0.04				0.04	—
GO-0.06				0.06	—
GO-0.08				0.08	—
GO–NS-0.02				—	0.02
GO–NS-0.04				—	0.04
GO–NS-0.06				—	0.06
GO–NS-0.08				—	0.08

Note: The number after GO or GO–NS is the content of GO or GO–NS.

**Table 4:** Number of freeze–thaw cycle resistance and typical damage patterns.

Lime Paste Type	Max. Number of Freeze–Thaw Cycles	Description of Typical Damage Patterns	Typical Damage Patterns
Pure sticky rice–lime paste	7	Corner flaking	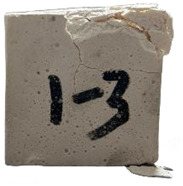
GO-0.02	10	Severe peeling	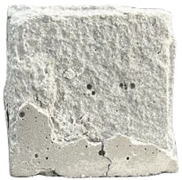
GO-0.04	13		
GO-0.06	14		
GO-0.08	12	Peeling, missing blocks	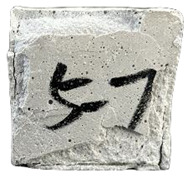
GO–NS-0.02	13	Cracks	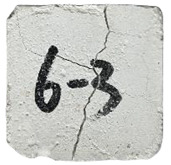
GO–NS-0.04	16	Seriously missing blocks	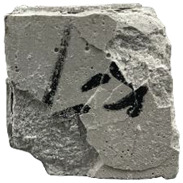
GO–NS-0.06	18		
GO–NS-0.08	16	Missing corner blocks, cracks	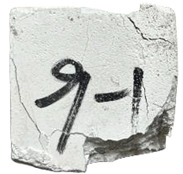

## Data Availability

Data will be made available on request.
